# Commercial Release of Genetically Modified Crops in Africa: Interface Between Biosafety Regulatory Systems and Varietal Release Systems

**DOI:** 10.3389/fpls.2021.605937

**Published:** 2021-03-22

**Authors:** Olalekan Akinbo, Silas Obukosia, Jeremy Ouedraogo, Woldeyesus Sinebo, Moussa Savadogo, Samuel Timpo, Ruth Mbabazi, Karim Maredia, Diran Makinde, Aggrey Ambali

**Affiliations:** ^1^Centre of Excellence for Rural Resources and Food Systems, Diran Makinde Center, African Union Development Agency-NEPAD, Ouagadougou, Burkina Faso; ^2^Centre of Excellence for Human Capital Institutions Development, African Union Development Agency-NEPAD, Nairobi, Kenya; ^3^Centre of Excellence for Rural Resources and Food Systems, African Union Development Agency-NEPAD, Dakar, Senegal; ^4^College of Agriculture & Natural Resources, Michigan State University, East Lansing, MI, United States; ^5^African Union Development Agency-NEPAD, Midrand, South Africa

**Keywords:** seed regulations, Africa, biosafety, GMOs (genetically modified organisms), variety registration

## Abstract

African countries face key challenges in the deployment of GM crops due to incongruities in the processes for effective and efficient commercial release while simultaneously ensuring food and environmental safety. Against the backdrop of the preceding scenario, and for the effective and efficient commercial release of GM crops for cultivation by farmers, while simultaneously ensuring food and environmental safety, there is a need for the close collaboration of and the interplay between the biosafety competent authorities and the variety release authorities. The commercial release of genetically modified (GM) crops for cultivation requires the approval of biosafety regulatory packages. The evaluation and approval of lead events fall under the jurisdiction of competent national authorities for biosafety (which may be ministries, autonomous authorities, or agencies). The evaluation of lead events fundamentally comprises a review of environmental, food, and feed safety data as provided for in the Biosafety Acts, implementing regulations, and, in some cases, the involvement of other relevant legal instruments. Although the lead GM event may be commercially released for farmers to cultivate, it is often introgressed into locally adapted and farmer preferred non-GM cultivars that are already released and grown by the farmers. The introduction of new biotechnology products to farmers is a process that includes comprehensive testing in the laboratory, greenhouse, and field over some time. The process provides answers to questions about the safety of the products before being introduced into the environment and marketplace. This is the first step in regulatory approvals. The output of the research and development phase of the product development cycle is the identification of a safe and best performing event for advancement to regulatory testing, likely commercialization, and general release. The process of the commercial release of new crop varieties in countries with established formal seed systems is guided by well-defined procedures and approval systems and regulated by the Seed Acts and implemented regulations. In countries with seed laws, no crop varieties are approved for commercial cultivation prior to the fulfillment of the national performance trials and the distinctness, uniformity, and stability tests, as well as prior to the approval by the National Variety Release Committee. This review outlines key challenges faced by African countries in the deployment of GM crops and cites lessons learned as well as best practices from countries that have successfully commercialized genetically engineered crops.

## Introduction

The development of genetically engineered crops (GMOs), categorized as genetically modified organisms (GMOs), follows a clear path, starting with gene discovery, followed by proof-of-concept (POC) studies, product development and deployment, and finally commercialization. Each plant regenerated from a single transformed cell carrying a gene of interest is referred to as an “event.” Hundreds of “events” are screened to select 2–3 promising events, called “lead events,” that can be commercialized. Similarly, the development of conventional crops through breeding follows a designated path, starting with the identification of core germplasms and traits or the creation of genetic variability by crossing or mutation, followed by segregation, successive backcrossing, and selection, as appropriate. The final product is a candidate cultivar or hybrid, which is subjected to multi-location testing to obtain approval for commercial cultivation, according to standard procedures of the respective jurisdictions. Upon the confirmation of its agronomic value for cultivation and registration, the variety passes through successive stages of seed increase (from breeders’ seed to foundation or basic seed) and quality management, eventually reaching the final commercial certified seed stage.

Both conventional and biotechnological approaches for seed development lead to the deployment of crops that contribute to food and nutritional security and socio-economic change. Moreover, both processes are regulated; genetically engineered crops are regulated by legislation within the Biosafety Act and Regulations, whereas conventional breeding is regulated by the Seed Act and pertinent regulations in each of the 55 member states of the African union. Many African countries require that new varieties be subject to National Performance Trials (NPTs), which involve the evaluation of certain aspects collectively known as DUS (distinctness, uniformity, and stability) as well as the evaluation of their cultivation value (Setimela et al., 2009). In addition to meeting DUS requirements, biotechnology-derived crops are subjected to food, feed, and environmental safety evaluations, prior to being approved as safe for human consumption and cultivation. Notably, in many countries, declaration of the safety of a biotechnology-derived crop is not equivalent to its approval for commercial use; instead, the approved events must be subjected to NPTs, with an accompanying evaluation of DUS parameters. It is also noteworthy that the regulatory agencies that approve the biosafety of biotechnology-derived crops and registration of seeds and cultivars cooperate with each other to commercialize genetically engineered crops.

The evaluation, registration, and commercialization of seeds are regulated under the Seed Act, and evaluation experiments are conducted either by or under the supervision of the National Performance Trial Committee (NPTC) and approved by the National Variety Release Committee (NVRC). However, after event approval by the national regulatory agency, it is critical that the NPTC and NVRC are convinced by the performance of the crop in the field or at least are confident in the data that the genetically engineered crops are now safe to go through NPTs and receive approval, just like conventional crops. In addition, the National Biosafety Authority (NBA) and other competent agencies should declare complete, but not partial, event deregulation/approval, and should fully entrust the NVRC and NPTC to conduct variety evaluation, release, and registration, without interference from the NBA. Experience in such matters in Africa has shown that this synchrony and harmony in decision making between the two regulatory entities has not been the case for the first wave of GM crops. In addition, there is a lack of clarity or guidelines on how NPTC and NVRC handle approved events, whereas the NBA and other agencies, under their mandate, completely approve the events. Event approval by the NBA is a requirement for variety registration. In this review, we aimed to report the experiences of selected African countries, and the steps that have been undertaken to counteract the impasse in the approval for the environmental release and commercialization of the GM improved seeds.

## Process of Varietal Release

**FIGURE 1:**
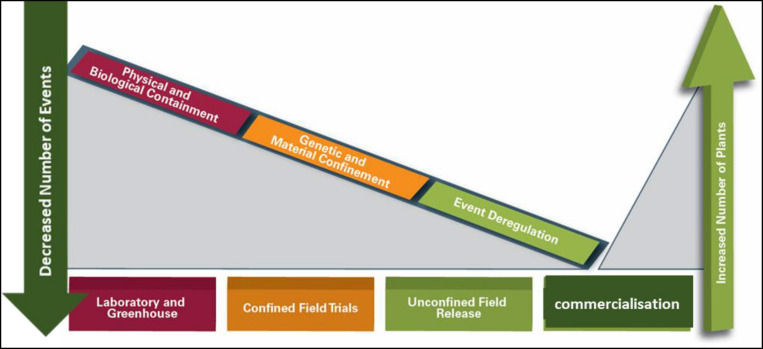
Biotechnology proof of concept, product development, and commercialization continuum and biosafety regulatory considerations.

The process leading to the commercialization of GMOs involves gene discovery, POC, product development, and general release or event approval (sometimes also referred to as event deregulation) (Figure 1).

### Gene Discovery

The development of a GM crop/product starts with gene discovery and gene function verification, which can broadly be categorized under functional genetics. Different functional genomics methods have been developed to study DNA, RNA, and proteins. DNA-based methods involve genetic interaction mapping (Gonatopoulos-Pournatzis et al., 2020), DNA–protein interactions (Cherstvy et al., 2008), and DNA accessibility or DNA-binding assays. RNA level-based approaches include the use of microarrays (Plomin and Schalkwyk, 2007), RNA sequencing (RNA-seq), serial analysis of RNA expression (Yamamoto et al., 2001), and massively parallel reporter assays (Melnikov et al., 2014; Kehe et al., 2019; Mulvey et al., 2020). Protein level-based techniques include the yeast-two hybrid mutation (Schwartz et al., 1998; Kondo et al., 2003) and deep mutation scanning, among other methods.

Although many genes and their functions have been discovered to date, only a handful have been incorporated into commercial GM crops currently on the market. There is great because of the disparity between gene discovery and the development of a commercially and economically viable product.

### Proof of Concept (POC)

The progression from gene discovery to lead event selection for product development is not simple or linear, but rather quite rigorous, time consuming, and punctuated by several modifications, such as promoter analyses, marker gene testing, and codon optimization, which may result in back-and-forth movement. This process of moving back and forth until achieving economically viable traits in lead events is referred to as POC. POC marks a stage during product development, where it is established that the product will function as intended (Collins English Dictionary, 2020) or where empirical evidence suggests that the genes under study confer the anticipated trait in the transformed events.

Before genes are assembled into constructs for transformation, they undergo a bioinformatic assessment to determine the safety of expressed proteins by assessing their allergenicity and toxicity potential. The successfully evaluated genes are assembled into a construct, which, in the simplest use of the term, consists of a promoter and structural genes, including a marker (reporter) gene and a transgene of interest. The construct is used to transform a crop of interest, and through several rounds of selection, hundreds to thousands of events are screened to identify promising events with the required gene expression. This work takes place in contained facilities, e.g., in a laboratory and/or a greenhouse. Biological and physical containment measures are employed during this step to ensure safety. After going through thousands of transformation events, 2–3 lead events are selected for further analysis.

### Product Development

Product development using biotechnological tools involves an assessment to ascertain that the product will function as intended and has commercial viability. In crops, traits of interest are usually incorporated into easy-to-transform varieties, with pre-established transformation and tissue culture regeneration protocols, and not directly into commercially viable varieties. In vegetatively propagated crops, such as cassava and banana, lead events constitute commercial varieties.

Product development is the process of designing, creating, and marketing new products or services to benefit customers. Sometimes referred to as “new product development,” this discipline is focused on developing systematic methods to guide all processes involved in launching a new product in the market. Creation of products with new or different characteristics provides additional benefits to consumers and end users. Product development may involve the modification of an existing product or its presentation, or the formulation of an entirely new product that satisfies a newly defined consumer need or market niche. Product development involves steps taken to conceive, design, and commercialize a product.

The term “product development” usually refers to the process of incorporating the traits into the final crop variety that will be commercially released and along with studies related to its biosafety. The product development method depends on the reproductive system of the crop: in self-fertilizing crops such as sorghum, lead events are introgressed into the farmer-preferred commercial cultivars, which are referred to as essentially derived varieties (EDV); in vegetatively propagated crops, the lead event is usually the final variety to be commercially released; in hybrids, the traits are incorporated into one of the inbred lines to be used for the development of the final hybrid. Product development also entails developing a regulatory package that consists of core and event-specific data (see section “Informal Seed Sector”). Product development is mainly conducted in field trials with genetic testing and material confinement. During product development, it is necessary to verify that gene expression is within the threshold of the purpose of the reason for the gene insertion. Studies must focus on confirming the intactness of the gene insert, exactness of the intended effects, gene stability and efficacy, and copy number of the insert.

**FIGURE 2:**
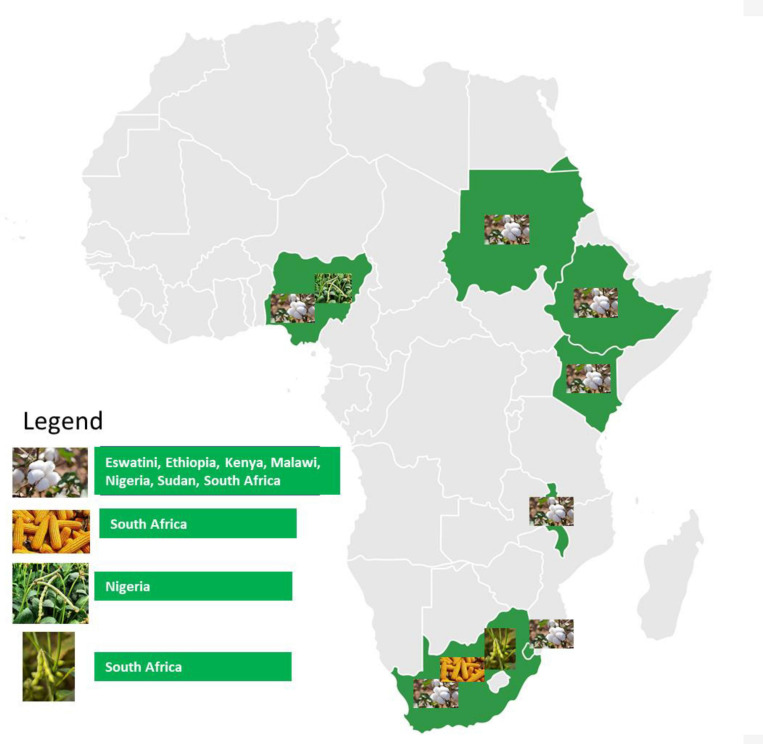
African Countries Status with Commercialized Biotechnology Crops.

In African countries, research, development, and deployment of biotechnology crops are at various levels of advancement with a few countries releasing commercial crops for farmer adoption (Table 1). Among factors that influence the speed of adoption of GM crops such as biosafety, public acceptance, political will and support is preponderant. In Kenya, the commercial release of the Bt cotton could be mainly attributed to the nation-wide need for cheap, locally procured raw materials to revitalize the textile industry. One of the four Kenya presidential pillars in his policy direction for his tenure in office is to spur economic development was enhancing Kenya Manufacturing Industry, of which textile industry was earmarked. Kenya in the scoreboard of the countries with the approval of Bt cotton in the continent, it joined the changed dynamics of the six countries who have already commercialized one or more GM crops (Figure 2).

**TABLE 1:** Status of Agricultural Biotechnology crops in Africa.

S/No	Country	Crop	Trait	Status
(1)	Burkina Faso	Cowpea	Insect resistance	CFT
(2)	Cameroon	Cotton	Insect resistance, herbicide tolerance	CFT
(3)	Ethiopia	Maize	Insect resistance, drought tolerance	CFT
(4)	Ghana	Rice	Nitrogen use efficiency, drought tolerance, salinity tolerance	CFT
		Cowpea	Insect resistance	CFT
		Maize	Insect resistance, drought tolerance	CFT
(5)	Kenya	Sorghum	Crop composition	CFT
		Potato	Disease resistance	CFT
		Cotton	Insect resistance	CFT
		Sweet Potato	Virus resistance	CFT
		Maize	Insect resistance, drought tolerance	CFT
		Maize	Drought tolerance	CFT
		Cassava	Disease resistance	CFT
		Banana	Disease resistance	CFT
(6)	Malawi	Cowpea	Insect resistance	CFT
		Banana	Virus resistance	CFT
(7)	Mozambique	Maize	Insect resistance, drought tolerance	CFT
(8)	Nigeria	Sorghum	Crop composition	CFT
		Cassava	Crop composition	CFT
		Cassava	Crop composition, disease resistance	CFT
		Rice	Nitrogen use efficient, drought tolerance, salinity tolerance	CFT
		Maize	Insect resistance, drought tolerance, herbicide tolerance	CFT
(9)	South Africa	Sugarcane	Crop composition	CFT
		Sugarcane	Drought tolerance, insect resistance, herbicide tolerance, nitrogen use efficiency	CFT
		Cassava	Crop composition	CFT
		Maize	Insect resistance, drought tolerance, herbicide tolerance	CFT
(10)	Tanzania	Maize	Insect resistance, drought tolerance	CFT
(11)	Uganda	Rice	Nitrogen use efficient, drought tolerance, salinity tolerance	CFT
		Potato	Disease resistance	CFT
		Maize	Insect resistance, drought tolerance	CFT
		Cassava	Disease resistance	CFT
		Banana	Insect resistance	CFT
		Banana	Disease resistance	CFT
		Banana	Crop composition	CFT

*^1^https://croplife.org/plant-biotechnology/innovation-in-plant-biotechnology/^2^https://www.ippmedia.com/en/news/ministry-cancels-gmo-seed-trials?s=03*

## Informal Seed Sector

The informal seed sector supplies approximately 80% of the seeds in sub-Saharan Africa. In 1970s and 1980s, the public sector seed programs targeted the dissemination of high-quality seed of improved varieties in sub-Saharan African countries, assuming that the informal seed system would disappear. While the I990s saw a decline in the role of the public seed sector in seed dissemination and led to active Private Seed Sector participations, the informal seed sector persisted (Niels and De Boef, 2012). This led to the concept of Integrated Seed Sector Development (ISSD) in Africa, which was initially formulated as a way to integrate formal seed systems and farmer-owned seed systems at the technical (Louwaars, 1996a) and institutional levels (Louwaars, 1996b; De Boef et al., 1997; Niels and De Boef, 2012). The key principle of ISSD is to facilitate interaction between the informal and formal seed sectors. The question pertinent to this paper is whether the informal seed sector can safely handle and deliver GMO seeds to farmers. This concern would be possible if intellectual property (IP) rights related to GMO seeds are provided as public goods, or if the farmers can maintain the integrity and safety of the GMO traits. Nonetheless, the informal seed sector will need to be backstopped by the public sector to ensure the genetic integrity of the GM crops through stewardship. In open-pollinated crops, which are developed into hybrids, it will be futile to let farmers save seeds beyond the first planting, as the genetic stability and integrity of the GM trait will deteriorate. This is not just specific to GM crops but also conventional hybrids because hybrid vigor decreases upon seed increase. Moreover, the dominant seed system development pathway promoted by international development is characterized by formalization and commercialization of the seed sector (Westengen et al., 2019). The inroads of improved seeds, particularly hybrid seeds, in farmer-designed seed systems reflects that these seeds are highly profitable for private seed companies, which discourages saving seeds from the previous harvest. However, in vegetatively propagated crops (such as cassava and banana) or self-pollinated crops (such as cowpea and rice), if the IP rights are negotiated to provide the technology as public goods, then there will be opportunities for the informal seed sectors to provide seeds to farmers. It will be important for the informal seed sector to obtain assistance from public seed sectors for monitoring and ensuring the stability of GM traits and the genetic background of the crop, and for replenishing the seed stocks. One example of such technology is the case of Bt cowpea in Nigeria, where SAMPEA 20-T was developed through a partnership that brought together Nigeria’s Institute for Agricultural Research (IAR), National Biotechnology Development Agency (NABDA), the Agricultural Research Council of Nigeria (ARCN), Australia’s national science agency (CSIRO), and the Danforth Plant Science Center. Bayer Crop Science provided the *Cry1Ab* (Bt) gene on a royalty-free humanitarian basis to ensure that the seeds are affordable to small-scale farmers. The partnership was coordinated by the African Agricultural Technology Foundation (AATF), with sustained funding by United States for International Development^1^.

## Outline of the Biosafety Regulatory Package

To conduct regulatory data development and approval, the identified lead event from the research and development phase of the product development cycle enters the regulatory assessment cycle for testing and approval to ensure its safety. Studies in the regulatory assessment cycle are conducted according to national and international guidelines established by a national biosafety regulatory agency. The regulatory data, which are generated in the laboratory, greenhouse, and field experiments during the assessment cycle, form the biosafety regulatory package. The purpose of the regulatory package is to provide adequate data for conducting risk analysis and for ensuring that the biotech-derived product is safe for human health, the environment, and animals. Two broad data classifications are analyzed: food and feed safety, and environmental safety. After 23 years of the commercialization of GM crops in 70 countries (ISAAA, 2018), the required biosafety data are quite standard, with only slight variation among countries. The guiding principles and framework outline that the constitution of food/feed safety and environmental safety data have been developed over time from several consultative workshops involving many renowned international agencies, including the Food and Agriculture Organisation (FAO) of the United Nations, World Health Organisation (WHO), Codex Alimentarius Commission, Organisation for Economic Co-operation and Development (OECD), and the European Food Safety Authority (EFSA).

The initial work regarding the safety of GM food and feed was first documented by the OECD (1993a, b), which established the concept of familiarity and substantial equivalence as guiding principles for assessing the safety of GM foods. The FAO/WHO consultative meetings of 1996 and 2001 expounded and concluded that substantial equivalence should be an important component in the safety assessment of foods and food ingredients derived from GM plants intended for human consumption (FAO, 1996; FAO/WHO, 2000). The 2000 FAO/WHO consultative meeting on foods derived from biotechnology held in Geneva, addressed all aspects of the safety of food derived from GM plants, focused on the applicability of substantial equivalence as a general guidance for scientific risk assessment, and agreed on the reliability of the decision-tree approach in the process of assessing the allergenicity of foods derived from GM crops and their novel proteins (FAO/WHO, 2001). FAO/WHO held several other consultative meetings on safety of food derived from recombinant DNA microorganisms (FAO, 2001b), evaluation of allergenicity (FAO, 2001a), recombinant DNA animals (FAO, 2007). Codex Alimentarius in the 23rd session of FAO/WHO in 1999 established an *ad hoc* intergovernmental task force on food derived from biotechnology to develop standards, guidelines, or recommendations. The Codex Alimentarius and the associated subsidiary bodies have reflected on the results of these consultations and developed a document on principles of risk assessment of GMO foods (CODEX, 2003c) and three guidelines for food and feed safety from recombinant DNA plants, microorganisms, animals^2^ (CODEX, 2003a, b, 2008) and revised second edition covering the three preceding guidelines (FAO/WHO, 2009).

Subsequently, the EFSA published several complementary scientific documents on food safety, with some containing guidelines for the risk assessment of GM plants (used for feed and food) (EFSA, 2006, 2011a, 2013), GM microorganisms (EFSA, 2011b), and GM animals (EFSA, 2012). Additionally, the EFSA provides guidelines for the allergenicity assessment (EFSA, 2017) of GM plants and microorganisms as well as that of the derived food and feed (EFSA, 2010a). Moreover, the EFSA performed a food and feed risk assessment from stacked events (EFSA, 2007), examined the role of animal feeding trials on the safety and nutritional assessment of GM plant-derived food and feed (EFSA, 2010a) as well as the role of animal feeding studies (EFSA, 2008), provided opinions on 90-day whole-food feeding trials in animals (EFSA, 2011c) and scientific opinions on statistical considerations for the safety evaluation of GMOs (EFSA, 2010b), and performed a literature review of *in vitro* digestibility tests for allergenicity (EFSA, 2010c, 2013) guidance of allergenicity (EFSA, 2017) and the applicability of a 90-day repeated-dose oral toxicity study (EFSA, 2014).

Other important documents include publications on crop biology and selective OECD guidelines for testing that could be selectively applied to GMO toxicological testing, such as 20-day OTG 406 repeated dose toxicological studies in rodents, and 90-day OTG 408 repeated dose oral toxicity studies in rodents as well as post-market monitoring (EFSA, 2012). The EFSA guidelines for food/feed safety assessment have largely been accommodated into EU legislation as an appendix to Implementing Regulation (EU) 503/2013, with the caveat that these guidelines render all recommendations legally binding. Moreover, implementing the regulation poses a mandatory requirement for 90-day feeding studies with new single events (only facultative in the EFSA guidelines). The afore-mentioned publications contributed to the framework and components of the food safety regulatory packages, which are currently sought by many countries for the evaluation and approval of the food safety regulatory package of GM food and feeds. These regulatory packages mainly consist of general considerations, host plant descriptions, the use of GM plants as food, descriptions of donor organisms and genetic modifications, safety assessment in terms of protein toxicity, amino acid sequence homology, and gastric and pepsin digestibility, in oral toxicity studies, allergenicity amino acid sequence comparisons, enzymatic degradation, heat stability, specific serum screening (i.e., immunological studies), and pepsin resistance.

### Regulatory Packages for Environmental Safety

Several concepts and guiding principles developed by the OECD and FAO for assessing food safety are applicable in environmental risk assessment (ERA); for example, comparator/substantial equivalence, familiarity, and the case-by-case approach (OECD, 1993a). The report published by the US Environmental Protection Authority (EPA, 1998) on ‘Ecological Risk Assessment’ contained some concepts that have been adopted for the ERA of GMOs. These concepts include a three-phased approach that consists of problem formulation, analysis, and risk characterization (EPA, 1998). The six ERA steps described in the Directive 2001/18/EC cover problem formulation include risk characterization, hazard characterization, exposure characterization, risk management strategies, overall risk evaluation, and conclusions (EC, Directive 2001/18/EC). It is generally believed that the ERA components will vary depending on the receiving environment, and therefore should be considered on a case-by-case basis. For example, the EFSA Scientific Panel outlines eight key areas of environmental risk for GMOs, as defined in the Directive 2001/18/EC and EFSA (2010c): (1) changes in plant fitness owing to genetic modification; (2) potential for gene transfer and its environmental consequences; (3) interactions between GM plants and target organisms; (4) interactions between GM plants and non-target organisms; (5) effects on animal and human health; (6) interactions with biogeochemical processes and the abiotic environment; (7) impacts of specific cultivation, management, and harvesting techniques; (8) risk management strategies (EFSA, 2012). The EFSA guidelines also cover composition analysis, expression analysis, and phenotypic analysis under ERA; however, in practical terms, the afore mentioned are categorized under food safety, although the data were collected from GM events grown in the field. For the most commercialized GM crops, ERA mainly comprises effects on non-target organisms, impact of gene flow, potential for invasiveness, and effects on soil microorganisms.

### Event Deregulation or General Release or Approval

Event deregulation involves the regulatory agencies of the recipient country, and the examination of the regulatory package to decide whether the GM crop is safe for release into the environment and for human consumption. This analysis is usually based on the representative lead event. After the lead event has been deregulated, the traits can be introgressed into local cultivars for commercial release, by producing EDVs. In many countries with seed and variety Acts, new commercial varieties cannot be released into the market until they have been subjected to NPTs and evaluated in terms of DUS. The following sections outline these requirements in African countries, which have recently commercialized GM crops, including Eswatini, Ethiopia, Kenya, Malawi, Nigeria, and Sudan; Mozambique and Ghana have yet to commercialize biotech crops.

## Overview of the Regulation of National Seed Sector Systems

### Regulation of the Seed Sector in Eswatini

In Eswatini, the seed sector is regulated by three pieces of legislation: (1) Plant Control Act, 1981, a phytosanitary legislation that regulates the movement and growth of plants to prevent the introduction of pests and diseases, and to harmonize and align the principles of the International Plant Protection Convention (Eswatini, 2000b); (2) Seeds and Plant Varieties Act of 2000, which regulates the requirements, procedures, and practices along the seed value chain (Eswatini, 2000a); (3) Plant Varieties Regulations, which describe detailed procedures and standards to be observed during seed certification and seed testing (Eswatini, 2000a).

#### Variety Evaluation, Release, and Registration in Eswatini

The Seeds and Plant Varieties Act, 2000 of Eswatini details procedures for the production of certified seeds and the recognition of varieties produced outside Eswatini. For certified seed production, the applicant provides DUS data and the results of the Value for Cultivation and Use (VCU) tests. The Act has a provision for the appointment of a VRC, whose role is to stipulate the evaluation criteria and deliberate on the results for the release of a variety (Eswatini, 2000b). The development of plant breeder rights legislation is ongoing, but procedures for the production and distribution of certified seeds for farmers are in place. A key example is the cooperation of the Eswatini Cotton Board with the Bt cotton technology developer and the biosafety regulator to facilitate the adoption of improved Bt cotton crops (ABNE, 2019).

#### Regulation of GMOs in Eswatini

GM crops in Eswatini are regulated by the Biosafety Act of 2012, which is also under review regarding the amendment of clauses on liability and redress, and the expansion of its scope to regulate emerging technologies, including genome editing. The GM crop approval procedure in Eswatini goes through a three-step process. First, the application is submitted to the Eswatini Environmental Authority, an independent and competent biosafety authority agency under the Ministry of Tourism and Environmental Affairs, which checks the completeness of the application. Second, the application is reviewed by technical experts from the National Biosafety Advisory Committee appointed by the Eswatini Environmental Authority, which provides a review report with the recommendations. Finally, the report is forwarded to the Biosafety Board to obtain a final decision on the application, i.e., either to approve with terms and conditions or reject with reasons; this is oversighted by the Minister of Tourism and Environmental Affairs (ABNE, 2019).

### Legal and Regulatory Framework of the Seed Sector in Ethiopia

The revised Seed Law of Ethiopia issued in 2013 as Proclamation No. 782/2013 (Federal Democratic Republic of Ethiopia [FDRE], 2013) replaced the former Seed Law issued as Proclamation No. 206/2000 in 2000. Article 17 (3) of the current Seed Law allows the introduction of GM seeds if approval on biosafety matters has been granted by the Environment, Forest, and Climate Change Commission, as indicated in Proclamation No. 655/2009 and Proclamation (Amendment) No. 896/2015. The Seed Legislation and Regulations need to be revised and harmonized with the International Seed Testing Association to facilitate seed imports and exports of diverse crop cultivars. Seed quality control and certification, which used to be conducted by the National Seed Industry Agency, are now undertaken by the Animal and Plant Health Directorate of MoARD. The establishment of the National Seed Quality Control and Certification Division under MoARD and BoARD alone is not a solution for the current seed quality problem.

#### Variety Evaluation, Release, and Registration in Ethiopia

In 1978, the Ethiopian government convened a National Seed Council (NSC) to consider the issue of seed supply. The National Crop Improvement Committee established the NVRCs in 1982. In Ethiopia, two steps are involved in the release of a new variety or a hybrid developed by a breeder: (1) testing the new improved variety, and (2) registering and releasing the variety. Both steps are undertaken by the NVRC. The NVRC is involved in testing and releasing varieties and hybrids, compiling the National Variety list, and preparing the registry book, which includes all crop varieties released in the country.

New varieties are tested for at least 2 years in regional or national trials at research stations in 3–5 locations, and are subjected to a 1-year on-farm trial (Alemu and Spielman, 2006; Setimela et al., 2009). The NVRC elects a technical subcommittee that oversees DUS compliance. The role of the NVRC is to review the DUS data and cultivation value of new hybrids or varieties for release, and to determine their approval and registration in the National Seed Variety Registry.

#### National Biosafety Systems in Ethiopia

The approval of Bt cotton in Ethiopia for environmental release and variety registration has been smooth and unique. The accelerated variety registration and commercial cultivation of Bt cotton in Ethiopia emanated from the imperative of the Ethiopian government to support the cotton sector for accelerating its development as a prerequisite for industrialization and job creation, while functioning as a springboard for economic transformation.

The normal procedure for testing and granting environmental release approval for GM crops in Ethiopia follows the provisions in the country’s Biosafety Proclamations (Proclamation No. 655/2009 and the Amendment included in Proclamation No. 896/2015). Once the biotech-derived seeds have passed the safety parameters specified in these Proclamations and the implemented Biosafety Directives, and have officially been granted approval for environmental release by the Environment, Forest, and Climate Change Commission, the materials undergo the process that conventional seeds are subjected to for registration and commercial cultivation, as specified in the Seed Proclamation (Proclamation No. 782/2013).

### Legal and Regulatory Framework of the Seed Sector in Ghana

In Ghana, the seed sector is regulated by the Plants and Fertilizer Act of 2010 (803) (Republic of Ghana [RoG], 2010). The new Act replaced the Plant Quarantine Act of 1965 and the National Redemption Council Decree 100 of 1972 (Alhassan and Bissi, 2006). The Act consists of three parts: part 1 refers to plant protection and regulates the sanitary and phytosanitary parameters of seed import and export to prevent the introduction of plant pests; part 2 regulates the testing, registration, and release of certified seeds; part 3 refers to fertilizer control and regulations.

#### Variety Evaluation, Release, and Registration in Ghana

Part 2 of the Plants and Fertilizer Act of 2010 (803) regulates the evaluation, registration, and release of varieties (Republic of Ghana [RoG], 2010) and is supported by draft Regulations. The Act establishes the NSC, the Technical and Variety Release Committee (TVRC), and the National Variety Release and Registration Committee (NVRRC) as bodies responsible for the regulation of the evaluation, release, and registration of varieties (Republic of Ghana [RoG], 2010); however, there is overlap in the membership and duties of the TVRC and NVRRC (Tripp and Mensah-Bonsu, 2013). The NSC formulates policies encompassing the development, production (standards and seed certification), and marketing of seeds (Republic of Ghana [RoG], 2010). The TVRC is established under the NSC, and its major functions are to act in an advisory capacity to the NSC regarding the prescribed seed standards and certification, the development of a national seed register, recommendation of new varieties for release and varieties to be removed from the register, and creation and update of the National Variety Register (Republic of Ghana [RoG], 2010). The NVRRC performs functions similar to those assigned to the TVRC, i.e., recommending varieties to the NSC for inclusion in the National Variety Register, and updating the register (Republic of Ghana [RoG], 2010). If a breeder wants to perform variety testing, they must provide the NVRRC with enough seeds for it to arrange field-testing and inspection (Tripp and Mensah-Bonsu, 2013).

A new variety will be entered into the National Variety Register after the DUS and cultivation value tests have been completed, in accordance with guidelines developed by the TVRC and published in the Regulations (Republic of Ghana [RoG], 2010). The NSC and TVRC will each include one member from the Biotechnology and Nuclear Agricultural Institute (Republic of Ghana [RoG], 2010).

#### Regulation of GMOs in Ghana

The enactment of the Biosafety Act 831, 2011 which *inter alia* established the NBA, resulted in significant progress in the creation of an enabling regulatory environment. The Act regulation contained use, release, importation, and placing in the market. Ghana adopted Biosafety Regulations on 28 June 2019, and now has full legal regime to receive and process all applications including those for environmental release (ABNE, 2019). Following the conclusion of field trials for pod borer resistant (PBR) cowpea, there are indications that an application for environmental release will be submitted to the Ghana NBA for permit. In addition, field trials for the NEWEST rice project resumed in 2019, after the construction of a rain shelter and the renewal of the permit in 2019 (ABNE, 2019).

### Regulation of the Seed Sector in Kenya

Important regulatory instruments pertinent to variety testing, registration, and release in Kenya are the Seed and Plant Varieties Act (Seed Act; Cap. 326) (GoK, 2012) and the Seeds and Plant Varieties Regulations (NPT Regulations) (GoK, 2009; Kuhlmann and Zhou, 2015). The Seed and Plant Varieties Act, Cap. 326, guides the regulatory process of seed release, certification, and production (Kuhlmann and Zhou, 2015). The Seeds and Plant Varieties Regulations established the NPTC and NVRC. The NPTC oversees the conduct of the NPTs, reviews the report, and makes recommendations to the NVRC. The NVRC considers the NPTC’s report (including DUS parameters), approves and releases qualifying varieties, and maintains the National Variety Register of approved varieties (GoK, 2009).

#### Variety Evaluation, Release, and Registration in Kenya

Variety release procedures usually consist of nation-wide performance tests and administrative registration procedures (Kuhlmann and Zhou, 2015). To officially release and register a new variety in Kenya, the following guidelines must be followed: the variety must (1) undergo NPTs for at least two seasons, (2) be superior in terms of yield or other special attributes, (3) be distinct, uniform, and stable (DUS) in terms of essential characteristics, (4) have a valid descriptor for seed certification, and (5) be approved and released by the NVRC (Sikinyi, 2010).

#### Biosafety Regulations for GM Crops in Kenya

In Kenya, the Biosafety Act 2009 and supporting Regulations provide biosafety approval for the commercialization of the genetically engineered Bt cotton. Some of the other crops currently in the pipeline for advanced confined field trial (CFT) testing prior to commercial release include Bt Maize MON 810 and drought-tolerant TELA maize MON 87460.

### Regulation of the Seed Sector in Malawi

The seed sector in Malawi is regulated under the Seed Act of 2005 and the recently published Seed Regulations 2018 (Republic of Malawi [RoM], 2005, 2018). The seed legislation is regulated by the Department of Agricultural Research Services (DARS) under the Ministry of Agriculture and Food Security, in accordance with the National Seed Policy of 1993 (Mloza-Banda et al., 2010). The Seed Services Unit of DARS is responsible for seed certification, quality control, and the operation of seed testing laboratories (Mloza-Banda et al., 2010). The Seed Services Unit performs these duties through seed crop registration, seed crop field inspections, seed sampling, laboratory seed testing, plot checking, seed monitoring, and farmer training (Mloza-Banda et al., 2010). Seed Regulations 2018 has been updated to conform to the seed standards set by the International Union for the Protection of New Varieties and the OECD (Republic of Malawi [RoM], 2018).

#### Variety Evaluation, Release, and Registration in Malawi

The VRC, which was once responsible for releasing crop varieties in Malawi, has been replaced by the Agricultural Technology Clearing Committee (ATCC). The ATCC consists of representatives of partners in agriculture, such as the DARS, Department of Crop Production, Department of Agricultural Extension Services, Agricultural Research and Extension Trust, Tea Research Foundation, Pesticide Board of Malawi, National Commission of Science and Technology, and University of Malawi. The Secretariat of ATCC is the DARS (Mloza-Banda et al., 2010). The DUS characteristics of certified seeds are evaluated before they are entered into the National Register of Cultivars and/or before their breeders are granted plant breeder’s rights. The VCU of seeds is assessed either for at least two seasons at a research station and one season on a farm with several sites per agroecology target or for three seasons at a research station and 2 years on a farm with few sites per agroecology target.

#### Biosafety Framework in Malawi

Malawi Government signed the Cartagena Protocol on Biosafety in May 2000 and ratified it in 2009, and then enacted the Biosafety Act in October 2002 under the Minister responsible for Environmental Affairs, followed by Biosafety Regulations in 2007, National Biotechnology and Biosafety Policy in 2008, giving the country a functional biosafety regulatory framework^3^ Today, Malawi has commercialized Bt cotton. The Biosafety Act provides an institutional framework to the regulatory body for its operationalization which consist of the following: National Biosafety Regulatory Committee, Reviewers, Inspector, and Biosafety Registrar. The first Bt cotton CFT were conducted in 2011^4^.

### Legal and Regulatory Framework of the Seed Sector in Mozambique

The seed sector of Mozambique is regulated under the 12/2013 Seed Regulation Decree, which establishes an advisory role for the National Seed Committee (NaSC) toward the Ministry of Agriculture and the Variety Registration and Release Committee (Anon, 2019). The 12/2013 Seed Regulation Decree regulates the testing, registration, and release of new varieties. The seed law of 2001 makes seed registration and DUS testing mandatory for all seeds marketed in Mozambique; this includes the possibility of registering traditional and local varieties (Grain, 2005).

#### Variety Evaluation, Release, and Registration in Mozambique

The Department of Seeds in the Ministry of Agriculture is responsible for seed company registration, varietal release, seed quality control, and seed lot certification. The Department oversees DUS evaluation and VCU testing of new varieties (ISSD Africa Brief, 2012). The Seed Department of the Ministry of Agriculture is responsible by law (184/2001) for seed production inspection, quality control, and certification, marketing control, processing plant and seed warehouse inspection, and seed import and export controls. The multi-stakeholder NaSC oversees the seed sector and validates decisions on variety registration and release. The NaSC sub-committee on the Variety Registration and Release Committee prepares the registration and release of varieties, which are then implemented by the Department of Seeds (ISSD Africa Brief, 2012).

#### Biosafety Systems in Mozambique

Concerning GMOs, Mozambique signed the Cartagena Protocol on Biosafety in May 2000 and ratified it in October 2002, while it established an inter-institutional working group (Grupo Inter-Institucional Sobre Bio-Segurança) in 2002 to serve as the National Biosafety Committee, and designated the Ministry of Science and Technology as the NBA. The Republic of Mozambique established a regulatory pathway for GMOs through an act of Decree no. 6/2007 (regulation), with an amendment in 2014 to allow the commercialization of GMOs. The legal framework for GMOs was enacted though the Decree adopted in 2007 which was revised (ABNE, 2019). The Decree allows the appointment of a diverse group of experts under Grupo Inter-Institucional Sobre Bio-Segurança to serve as an advisory committee to the Minister of Science and Technology, Higher and Technical Vocational Education, which is a competent authority on matters pertaining to GMO approvals (ABNE, 2019). The decree also empowered the Minister to constitute an *ad hoc* Scientific Advisory Committee (ABNE, 2019).

### Regulation of the Seed Sector in Nigeria

The Seed Sector in Nigeria is regulated under the NSC Act of 2019 (Federal Republic of Nigeria [FERN], 2019). The law made provision for the NSC to regulate all aspects pertaining to the seed industry, including the regulation and control of seed testing as well as the registration and release of certified seeds. The Act repeals the National Agricultural Seeds Act, N5 Laws of Nigeria, 2004.

The National Agricultural Seed Act 71 of 1992 was reviewed by the National Seed Law Committee once in 2004 (National Agricultural Seeds Act 2004) and again in 2019 (National Agricultural Seed Council Act 2019) (Federal Republic of Nigeria [FERN], 2019). The NSC of Nigeria is the designated governing body mandated under the National Agricultural Seeds Act No. 72 of 1992 to analyze and propose programs, policies, and actions regarding seed development and the seed industry in general, including legislation and research on issues relating to seed testing, registration, release, production, marketing, distribution, certification, quality control, supply, and use, to regulate the import and export of seeds, and to control the quarantine regulations, among other functions (NASC, 2019). The NSC of Nigeria controls, supervises, and approves the activities of the following committees, among others, established by or pursuant to the decree: Crop Variety Registration and Release Committee, Seeds Standards Committee, and Seed Industry and Skill Development Committee (Kormawa et al., 2015; NASC, 2019).

#### Variety Evaluation, Release, and Registration in Nigeria

Decree No. 330 of 1987 promulgated the establishment of the National Crop Varieties and Livestock Breeds Registration and Release Committee, which works together with technical subcommittees. The activities of the National Crop Varieties and Livestock Breeds Registration and Release Committee and the technical subcommittees are coordinated by the National Centre for Genetic Resources and Biotechnology. The functions of the National Crop Varieties and Livestock Breeds Registration and Release Committee and the technical subcommittees, as spelt out by the decree, are to register and release superior crop varieties, livestock breeds, and fish strains to farmers and the agro-industries (NCGRB, 2016).

Nigeria made history in the commercial release of insect-resistant cowpea, a major food security crop, in 2019. The National Varietal Release Committee approved Sampea 20-T for registration and commercial release, and its seeds can be made available to farmers. Sampea 20-T is the first GMO cowpea variety in the world. This variety was developed through multiple partnerships; private sector donated the Bt genes; CSIRO of Australia provided the technology for transforming cowpea; CSIRO in collaboration with NARS of Ghana, Burkina Faso, and IAR of Nigeria were involved in product development; AATF played the role of bringing the partnership together and negotiating for IPR transfer. Field studies of these varieties confirmed the near complete protection against the pod borer (see footnote 4).

#### Biosafety Systems in Nigeria

Nigeria enacted a biosafety law, the National Biosafety Management Agency Act 2015, which mandates the National Biosafety Management Agency (NBMA) to, among other objectives, provide a holistic approach for the regulation of GMOs. An NBMA amendment bill was initiated by the House of Representatives in 2018 and passed as a law by the 8th National Assembly in 2019. GMOs are regulated by two agencies in Nigeria: NABDA, and NBMA. The NABDA mandate focuses on biotechnology policy, while the NBMA mandate focuses on the biosafety regulations of biotechnology-derived products. The NBMA was established in 2015 by an Act under the Federal Ministry of Environment. In the value chain of GMO product to be release into the environment or to the farmers, the NBMA receives the application and confirm the completeness of the document in accordance with the Cartagena protocol on biosafety, sets up a review process of the application with a timeline, and appoints technical experts. Then, the technical experts make recommendations to the Director General and the Chief Executive Officer of the Agency, who either approve or reject the application. The unique process of the Nigerian regulatory system is that the power of approval/rejection is vested on the Director General and the Chief Executive Officer, not the Minister under which the agency is supervised, as observed in other countries. The approval document is part of the report that accompanies the application for general release through the crop variety registration and release committee. Subsequently, the breeder seeds are ready for multiplication to generate certified seeds for distribution to the farmers. Nigeria is a case in point where the NBMA was called to the varietal release committee meeting to explain the procedures followed for the approval of Bt cotton and to reassure the committee about its safety for cultivation.

### Regulation of the Seed Sector in Sudan

Sudan enacted a new Seed Law in 2009. This law was subsequently expanded to enhance the development of the private seed industry in the country. According to this Law, the NSC oversees seed certification standards, and the Seed Administration of the Ministry of Agriculture is the national seed authority responsible for seed certification, quality control, and phytosanitation.

#### Variety Evaluation, Release, and Registration in Sudan

In Sudan, plant breeders conduct variety performance trials aiming to assess the VCU, while no DUS testing is required. The trials are conducted for a minimum of two seasons at two locations in the targeted region, and performance data are collected to determine if the variety can be released. The procedure and requirements to be followed by the applicants for the release of new varieties are issued by the VRC. The data are submitted to the VRC, and a decision is made based on the VCU performance data provided by the breeder. The breeder must provide a clear description of the variety to facilitate easy identification and verification. Varietal registration is performed. The VRC-approved varieties are included in the National List of Varieties for the recommended ecological regions. The new seed law also establishes the Seed Administration acts as a secretary for the NSC.

#### Regulation of Biosafety in Sudan

Sudan ratified the Cartagena Protocol on Biosafety in 2005 and developed a National Biosafety Policy in 2005. Subsequently, Sudan passed the Biosafety Law in June 2010, which led to the establishment of the Sudan National Biosafety Council (SNBC) in June 2012^5^.

## Regional Harmonization of Seed Legislation in Africa

Two approaches can be adopted for the harmonization of seed legislation: harmonization to conform to international best practices, or regional harmonization (Kuhlmann, 2015). Harmonization to conform to international best practices facilitates and promotes the modern and competitive seed industry (Gisselquist, 2001). Although regional harmonization has many facets, its objective is to create a large open market in place of many small national seed markets (Gisselquist, 2001). Five pertinent areas identified for the harmonization of seed laws, regulations, and standards include variety evaluation, release, and registration, seed certifications, sanitary and phytosanitary certifications, plant variety protection, and seed laws and regulations (ASARECA, 2004; Kuhlmann, 2015). Regional harmonization removes costly national seed laws, standards, and regulations as well as small national seed markets that create an unattractive environment for the local and international seed companies. One of the advantages of regional harmonization is improved farmer accessibility to improved high-quality seeds (ASARECA, 2004). Several African Regional Economic Communities have made commendable progress in the regional harmonization of seed laws and regulations in Africa, including the Economic Community of West African States (ECOWAS), Common Market for Eastern and Southern Africa (COMESA), East African Community (EAC), Southern African Development Community (SADC), Contre la Se’cheresse dans le Sahel (CILSS), West African Economic and Monetary Union, and the Association for Strengthening Agricultural Research in Eastern and Central Africa (ASARECA), although these communities are not considered as Regional Economic Communities.

### ECOWAS Seed Harmonization

In May 2008, the Ministers of ECOWAS countries approved Regulation C/REG.4/05/2008 on the harmonization of rules governing the quality control, certification, and marketing of plants (ECOWAS, 2008; Kuhlmann and Zhou, 2016). The West and Central African Council for Agricultural Research was tasked with implementing the ECOWAS Regulations. The West and Central African Council for Agricultural Research issued an official request to ECOWAS, the West African Economic and Monetary Union, and the Permanent Inter-State Committee for Drought Control in the Sahel member states, requesting the publication of the ECOWAS regulations in the official national gazettes of African countries, to allow the enforcement of the ECOWAS seed regulation across different regions (Kuhlmann and Zhou, 2016). The regulation requires that the West African Catalog for Plant Species and Varieties covers all varieties registered in the national plant variety catalogs of member states (ECOWAS, 2008; Kuhlmann and Zhou, 2016). This regional catalog is essentially a compilation of the national catalogs of individual countries (Keyser, 2013). Consequentially, varieties entered in the West African Catalog for Plant Species and Varieties can be traded, permitted, and multiplied throughout the region without the need for further registration. This contrasts the situation in COMESA and SADC regions, where a variety must be registered in at least two countries to be eligible for entry into the regional catalog.

The harmonized regulation of ECOWAS provides a clear diversion from the National Seed Certification Regulation. Sampling is carried out in accordance with the international rules developed by the International Seed Testing Association, which developed procedures for the field testing of seed quality to ensure uniformity in seed quality worldwide. The harmonized regulation of ECOWAS states that the national seed testing laboratories of member states, accredited by the International Seed Testing Association, will be authorized to issue an international certificate. In addition, the harmonized regulation does not require DUS and VFC testing for seed certification; instead, it classifies certified seeds based on the OECD classification system, which includes the following categories: first-generation or R certified seed, developed from basic seeds; second-generation or R2 certified seeds, developed from R certified seeds; third-generation or R3 certified seeds, developed from R2 certified seeds (ECOWAS, 2008).

#### ECOWAS and Harmonized Biosafety Regulations

ECOWAS constitutes a 15-member country regional grouping with a mandate of promoting economic integration in all fields of activity including industry, transport, telecommunication, energy, agriculture, natural resources, commerce, monetary and financial issues, and social and cultural matters (ECOWAS, 2008). Currently, ECOWAS is drafting Regulation C/REG.5/05/08 on the adoption of an Action Plan for the Development of Biotechnology and Biosafety in the ECOWAS Region.

### COMESA Seed Harmonization

COMESA comprises 21 African nations governed by the COMESA Treaty. The current strategy of COMESA is to ensure ‘economic prosperity through regional integration,’ and one of the approaches used to achieve this goal is to offer a wider, harmonized, and more competitive market to the 21 current member states. In March 2008, the COMESA Council of Ministers tasked COMESA with rationalizing and harmonizing the seed regulations and policies of member states (COMESA, 2014). The task undertaken by COMESA, the Alliance for Commodity Trade in Eastern and Southern Africa, and the African Seed Trade Association consummated in the finalization of the COMESA Seed Trade Harmonization Regulations 2014, which went into force in February 2014 after being approved by the COMESA Council of Ministers in Kinshasa (COMESA, 2014). Each COMESA member state is required to implement these COMESA Seed Trade Harmonization Regulations 2014 domestically, either by the adoption of the regulations in the countries where they are not currently implemented, or by adapting existing regulations to bring them into compliance and ensure their enforcement (COMESA, 2014). By December 2018, 7 of the 21 COMESA countries (Burundi, Kenya, Malawi, Rwanda, Uganda, Zambia, and Zimbabwe) had harmonized their National Seed Regulations with the Regional Seed Trade Regulations (COMESA, 2018).

These regulations cover seed certification, variety release, and sanitary and phytosanitary requirements. Varieties to be released shall be subjected to the DUS test, which would be carried out in accordance with the International Union for the Protection of New Varieties guidelines and the VCU or NPTs. In addition, the Regulation established a COMESA Seed Coordination Unit, a COMESA Seed Committee, and a COMESA Variety Catalogue (COMESA, 2014). Varieties tested at the national level can be included in the COMESA Variety Catalogue if they conform to the prescribed COMESA DUS and VCU tests (COMESA, 2014). The harmonized COMESA Seed Regulation also prescribes the use of ISTRA Seed Testing Methodologies for assessing the seed quality.

#### COMESA and Harmonized Biosafety Regulations

In 2014, the Council of COMESA Ministers endorsed the implementation of the COMESA Biotechnology and Biosafety Policy, which translated into the COMESA Biotechnology and Biosafety Policy Implementation Plan; this Plan seeks to increase investments in biotechnology applications and agricultural commodity trade in the region. At its onset, COMPIT is set to provide the states with a framework and mechanism for the regional risk assessment of GMOs intended for commercial planting, trade, and emergency food aid (COMESA, 2018). Thirteen COMESA countries have a biosafety regulatory system; six of these countries (Ethiopia, Sudan, Kenya, Malawi, Nigeria, and Eswatini) have commercialized GM crops (ABNE, 2019). The Article 132 of the COMESA Treaty requires member states to harmonize their policies and regulations, without impeding the export of crops, plants, seeds, and other products^6^. It is expected that the adoption of harmonized policy will allow GMO data transportability among COMESA members, notwithstanding respecting each member state sovereignty.

### SADC Seed Harmonization

In February 2010, the SADC Ministers of Agriculture signed a Memorandum of Understanding for the implementation of the SADC Harmonized Seed Regulatory System [Centre for Applied Legal Research (CALR), 2012], which supports measures necessary to facilitate the movement of seeds (as a commodity) between countries in the SADC region. For the system to be functional, it requires SADC member states to align their national seed regulations to the common standards, rules, and procedures outlined in the SADC Harmonized Seed Regulatory System. Countries implementing the SADC Harmonized Seed Regulatory System on a pilot basis are at different stages of the national policy alignment. Mozambique, Eswatini, and Tanzania have made significant progress, attributable to the official anchorage of the seed policy alignment process within their respective Ministries of Agriculture. With political support and the official government buy-in established, the role of the task teams in these countries is to concentrate on devising and implementing advocacy mechanisms aimed at hastening reform activities in and through each of the policy reform steps. Malawi, Zambia, and Zimbabwe have undertaken significant activities related to the SADC Harmonized Seed Regulatory System but have not yet managed to anchor the policy alignment process in their respective Ministries of Agriculture. As such, these three countries have yet to officially commence the policy alignment process according to the standard reform processes defined by their respective governments [Centre for Applied Legal Research (CALR), 2012]. Given the divergent views toward GMOs in SADC members states, the harmonization of GM regulation is predicted to be highly challenging. In 2003, SADC sub-regional level guidelines were drafted and adopted through the SADC Advisory Committee on Biotechnology and Biosafety. However, little or no progress toward implementation of the policy has been accomplished (Morris, 2014). Despite this slow progress, Eswatini, Malawi, Mozambique, South Africa, and Tanzania have made giant strides toward the application of biotechnology tools.

## Interface Between General Release, Variety Evaluation and Registration

Since a long time, African countries with Seed Acts have been effectively and efficiently evaluating and releasing conventionally bred cultivars. The role of the NVRC has been to register and release superior varieties for commercialization to benefit farmers. However, the advent of biotechnological tools as a new source of improved varieties to be evaluated and released through the Seed Act and Implementing Regulation mechanism presents a new challenge to the regulatory systems. This is because in this new approach the Seed Acts Regulators and Biosafety Act Regulators need to interphase their regulatory and decision-making processes to achieve complementary objectives of providing safety and improved seeds to farmers. In some African countries, different bodies regulate the seed sectors, and most of the institutions are government-based, which conduct research and provide seeds as a public good. The development of GM seeds tends to be dominated by the private sector, and because of high cost involved in the development of improved varieties, the seed have to be sold at some cost to recuperate the cost incurred. This is a challenge in African countries where all the research investment for a new variety is deemed as a public goods. However, it is important to note that many African countries are also involved in the supply of improved conventional crops, in which case the use of commercial seeds is not new (Table 2). In countries where GMO crop products have been approved as a variety, the biosafety clearance certificate was issued by the biosafety authority prior to the product advancement to the NPT for varietal registration according to the seed Act of the country.

**TABLE 2:** The biosafety regulatory framework and seed laws of selected African Countries.

	Biosafety regulatory framework	Seed acts and implementing regulations
**Kenya**		
Laws and Regulations	Biosafety Act 2009 and Implementing Regulations to cover contained use, environmental release, import, export, and transit	Seed and Plant Varieties Act (Seed Act; Cap. 326) (GoK, 2012) and the Seeds and Plant Varieties Regulations (NPT Regulations)
Agencies/Department	National Biosafety Authority is the Competent Authority	KEPHIS, Ministry of Agriculture
Committees	Scientific Advisory Committee	National Performance Trial Committee National Variety Release Committee
Nigeria		
Laws and Regulations	National Biosafety Management Agency Act 2015 revised in 2019 to National Biosafety Management Agency Act 2019	National Agricultural Seeds Act, N5 Laws of Nigeria, 2004 revised to give Nation Seed Act (NSC) Act 2019
Agencies/Department	National Biosafety Management Agency (NBMA) is the National Biosafety Authority	
Committees		National Crop Varieties and Livestock Breeds Registration and Release Committee
**Eswatini**		
Laws and Regulations	Biosafety Act of 2012 (under review)	Plant Control Act, 1981 (under review); Seeds and Plant Varieties Act of 2000 and Plant Varieties Regulations
Agencies/Department	Eswatini Environmental Authority,	
Committees	National Biosafety Advisory Committee	National Variety Release Committee
**Ethiopia**		
Laws and Regulations	Biosafety Proclamations (Proclamation No. 655/2009 and the Amendment into Proclamation No. 896/2015).	Seed Proclamation (Proclamation No. 782/2013) revised to give Proclamation No. 206/2000 in 2000
Agencies/Department	Environment, Forest, and Climate Change Commission,	National Seed Quality Control and Certification Division under MoARD
Committees		National Crop Improvement Committee
**Ghana**		
Laws and Regulations	Biosafety Act 831, 2011 and Implementing Regulations	Plants and Fertilizer Act of 2010 (803)
Agencies/Department	National Biosafety Authority	National Seed Council (NSC),
Committees		Technical and Variety Release Committee (TVRC), National Variety Release and Registration Committee (NVRRC)
**Malawi**		
Laws and Regulations	Biosafety Act was passed in 2002 and Implementing in 2007 and National Biotechnology and Biosafety Policy was enacted in 2008	Seed Act of 2005 and recently published Seed Regulations 2018
Agencies/Department	National Biosafety Regulatory Committee (NBRC) is the Competent Authority	The Seed Services Unit of DARS Department of Agricultural Research Services (DARS)
Committees		Agricultural Technology Clearing Committee (ATCC
**Mozambique**		
Laws and Regulations	Decree no. 6/2007 (regulation) with an amendment in 2014 to allow for the commercialization of GMOs to give Decree 71/2014 of 28 November 2014	12/2013 Seed Regulation Decree
Agencies/Department	Minister of Science and Technology, Higher and Technical Vocational Education, is competent authority on matters pertaining to GMO approvals	National Seed Committee (NaSC) in Ministry of Agriculture and the Variety Registration and Release Committee
Committees	The Grupo Inter-Institucional Sobre Bio-Segurança, (GIIBS) serve as an advisory committee to the Minister of Science and Technology, Higher and Technical Vocational Education	Department of Seeds in the Ministry of Agriculture
**Sudan**		
Laws and Regulations	Biological Safety Act 2010	New Seed Law in 2009
Agencies/Department	Sudan National Biosafety Council (SNBC)	National Seed Council
Committees		

Although Eswatini, Malawi, Ethiopia and Kenya recently commercialized Bt cotton from the private sector, and all have Seed Laws and regulations, the efficacy of different steps, starting from event approval to seed registration and commercialization, was not identical. For example, in Eswatini, the applicant submitted the Biosafety regulatory dossier to the competent authorities for event approval. The essentially derived GMO hybrids were subjected to two or three planting seasons in confined multilocation field trials to evaluate their agronomic performance and trait efficacy in the hybrid background. The Cotton Board Authority applied for the seed and made them available to the farmers in the country.

Malawi approved Bollgard II cotton event for environmental release in 2016, after conducting confined trials under the supervision of the NBA. Thereafter, four EDVs underwent NPTs in five locations were conducted in 2017 and 2018, after variety registration and commercial release were applied. However, given the controversy surrounding GM technology, this process has not been smooth. Several consultations and confidence-building meetings, including field visits, study tours, and consultative workshops, were conducted for key stakeholders to enhance the understanding and knowledge of decision making. Subsequently, the National ATCC approved the registration of the hybrids in 2019.

In 2019, the commercialization of Bt cotton in Kenya was a critical step because, in Africa, only Burkina Faso, Sudan, Nigeria, Eswatini, Malawi, and South Africa had commercially released GM crops. In 2016, the NBA of Kenya reviewed the application for the general release of Bt maize Mon810 and Bt cotton in January 2016. Upon request by Kenya NBA, the AUDA-NEPAD and Program for Biosafety Systems of IFPRI provided technical support to Kenya NBA Board in risk Assessment and decision-making pertinent to the GMO application. This was necessary because although NBA is competent on this subject matter and often uses external professional reviewers for the assessment and determination of biotechnology applications, the NBA Board is the final entity that determines the approval of GM crops in Kenya.

**FIGURE 3:**
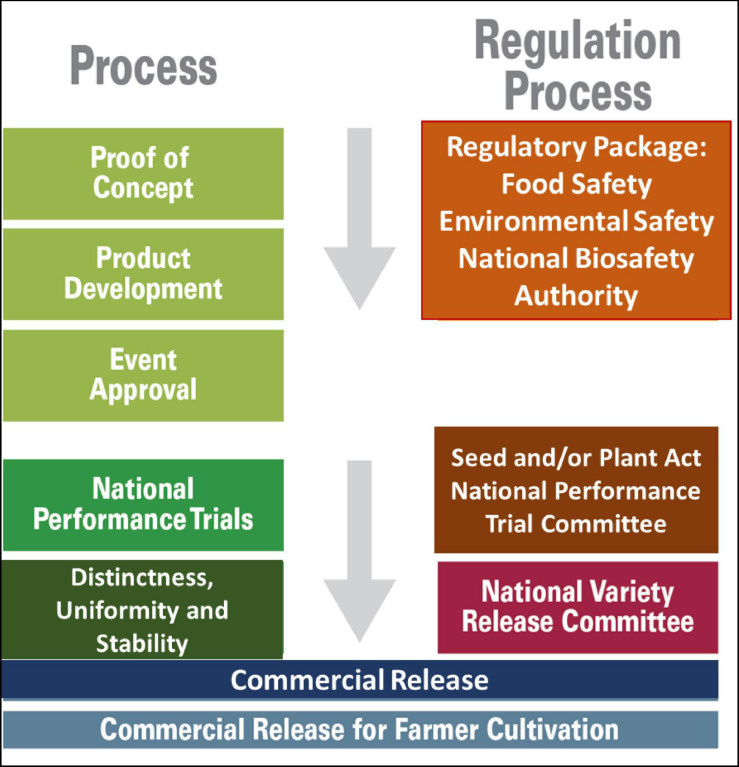
The pathway of the development of regulatory approval and commercial release of the genetically modified seeds.

The Technical Evaluation Report issued by the NBA recommended that the Bt maize event Mon810 and Bt cotton should undergo NPTs under the supervision of the NPTC of Kenya Plant Health Inspectorate Services before being considered for commercial release (Figure 3). Subsequently AUDA-NEPAD-ABNE convened an information sharing workshop between the NBA, NVRC, and NPTC. The purpose of the workshop was for NBA to familiarize itself with the regulatory requirements of the NVRC and NPTC for variety testing, registration, and release. This was because the processes of variety release and event deregulation (general release) are regulated by two government acts and regulations: Biosafety Act 2009 and Implementing Regulations. The release of GM crops is regulated by the Seed and Plant Varieties Act Cap. 329 subsidiary Seed Plant Varieties Regulation 2009 of Kenya, which also regulates the release of conventional crop varieties (GoK, 2009). The AUDA-NEPAD-ABNE-convened workshop resulted in a harmonized and synchronized regulatory decision regarding Bt cotton. After a successful NPT evaluation, the Bt cotton EDVs were registered, gazetted, and officially released by the NVRC under the auspices of the Ministry of Agriculture. Although AUDA_NEPAD-ABNE and partners such as PBS focused on the regulatory aspects to leverage regulatory approval, many other partners such as ISAAA and OFAB focused on creating awareness and leveraging on getting policy and political support for the technology.

In Ethiopia, two Bt cotton hybrids, JKCH1050 and JKCH1947, went through two seasons of CFTs under the supervision of the Biosafety Affairs Directorate of Ministry of Environment, Forest and Climate change. In 2018, the Ministry of Environment, Forest, and Climate (MEFCC) approved the environmental release of these hybrids. The approval was based on risk assessment and 2-year field evaluation data, which were used to determine performance under Ethiopian environmental conditions. There was not many institutional interfacings between the Biosafety regulatory and variety approval and registration, perhaps because of strong policy support, which focused on using cotton to revive the textile industry of Ethiopia.

Nigeria commercialized two cotton Bt cotton varieties, MRC 7377 BG 11 and MRC 7361 BG 11, in 2018. In May 2016, the NBMA approved for the Commercial Release and Placing on Market of the GM cotton event Mon 15985. Subsequently, the National Committee on Naming, Registration, and Release of Crop Materials (NCNRRCM) approved Bt cotton varieties, MRC 7377-BGII and MRC736-BGII, for commercial release in 2018. The Nigerian approach was exemplary because the NBMA approved the GMO event, and the NCNRRCM approved the variety registration and commercial release.

## Discussion

The biosafety approval, variety registration, and commercial release interface should be aligned with the NBA regulatory event approval, the NPTs, and the final variety release by the NVRC. The transition between regulatory agencies is not automatic, and capacity-strengthening workshops should be conducted to enable each entity to understand the roles and responsibilities of the other entities. The NBA should provide complete biosafety event approval covering food, feed, and environmental safety. The NPTC should develop guidelines for conducting NPTs of approved events, covering DUS and VCU testing, and should not be concerned with food, feed, and environmental safety data, as these aspects are covered by the NBA. The biosafety regulatory authorities and varietal registration and release authorities need to cooperate with each other for the efficient commercialization of GM crops so that millions of farmers and consumers in Africa can benefit from the safe products derived through modern biotechnology.

The biosafety authority should adhere to its mandate, which is to approve the event after all of the submitted safety data have been examined, and a determination has been made. The NPTC and NVRC should conduct/supervise the DUS and VCU tests on approved events that have been declared by the NBA as safe for human consumption and environmental release. In the African countries discussed in this review, including those that have approved GM crops for commercial release (Eswatini, Ethiopia, Kenya, Malawi, Nigeria, and Sudan) as well as those with a product that is near approval for commercial released (Ghana and Mozambique), it is imperative that the institutional mandate of each legal instrument should be respected and be adherent to without blocking the progress made in the safe adoption of biotechnology products in these countries.

The harmonization of Seed and Biosafety Regulations in Regional Economic Communities, such as SADC, COMESA, and ECOWAS, is important for the future facilitation of the regulatory approval of biotech crops on a regional basis and in a more cost-effective manner. The ongoing harmonization of biosafety regulations needs to be completed, while the more advanced harmonization of Seed Regulations needs to be operationalised. These two types of harmonizations will work in tandem to facilitate the safe regulation and approval of biotech crops in the region.

The role of political support in the determination of the sovereign decisions of a country on the safety and potential commercialization of biotech crops cannot be overemphasized. It is important that policy makers are provided with science-based information so that countries can reach sovereign decisions, in terms of biosafety, and achieve the safe approval of GM crops in the region, while ensuring environmental and human safety. In this regards, the interface between biotechnology regulation is that safety data presented to the regulatory institute should be trusted for event approval, and the superior performance of the crop for varietal registration should be within the role of the mandated institution, and enhances the responsibility of the separation rules.

## Author Contributions

All authors made substantial contributions to designing the review and acquiring relevant data. OA and SO wrote the first draft of the manuscript, while JO, WS, MS, ST, RM, KM, DM, and AA revised the manuscript critically and provided important intellectual content. All authors approved the final manuscript for publication.

## Conflict of Interest

The authors declare that the research was conducted in the absence of any commercial or financial relationships that could be construed as a potential conflict of interest.

## Footnotes

1 https://geneticliteracyproject.org/2020/06/02/landmark-approval-of-gmo-bt-insect-resistant-cowpea-leads-nigeria-toward-sustainable-farming/

2 see http://www.fao.org/3/a-a1554e.pdf

3 2014 Third year Bt cotton cultivation in Sudan. Sudan Bt Cotton Booklet (Web Version) (www.isaaa.org).

4 https://geneticliteracyproject.org/2020/06/02/landmark-approval-of-gmo-bt-insect-resistant-cowpea-leads-nigeria-toward-sustainable-farming/

5 2014 Third year Bt cotton cultivation in Sudan. Sudan Bt Cotton Booklet (Web Version) (isaaa.org). Sudan commercialized the first Bt crop in 2012.

6 http://africaleadftf.org/wp-content/uploads/2016/09/COMESA-Seed-Harmonisation-Implementation-Plan-COM-SHIP-_JULY-2014.pdf

## Abbreviations

AATF: African Agricultural Technology Foundation
ABNE: African Biosafety Network of Expertise
ARCN: Agricultural Research Council of Nigeria
ATCC: Agricultural Technology Clearing Committee
ASARECA: Association for Strengthening Agricultural Research in Eastern and Central Africa
Bt: *Bacillus thuringiensis*
CSIRO: Commonwealth Scientific and Industrial Research Organisation
COMESA: Common Market for Eastern and Southern Africa
CILSS: Contre la Se’cheresse dans le Sahel
CFT: confined field trial
DARS: Department of Agricultural Research Services
DUS: distinctness, uniformity, and stability
EAC: East African Community
EDV: essentially derived varieties
EFSA: European Food Safety Authority
ERA: environmental risk assessment
EU: European Union
FAO: Food and Agriculture Organisation of the United Nations
GM: genetically modified crop
GMOs: genetically engineered crops
IAR: Institute for Agricultural Research
LMOs: living modified organisms
NABDA: National Biotechnology Development Agency
NaSC: National Seed Committee in Mozambique
NBA: National Biosafety Authority
NBMA: National Biosafety Management Agency
NPT: national performance trial
NPTC: NPT Committee
NSC: National Seed Council
NVRC: National Variety Release Committees
NVRRC: National Variety Release and Registration Committee
OECD: organization for economic Co-operation and development
POC: proof of concept
SADC: Southern African Development Community
SARI: Savannah Agricultural Research Institute
TVRC: Technical and Variety Release Committee
VCU: value for cultivation and use
USAID: Unite State Agency for International Development
VRC: Variety Release Committee
WHO: World Health Organisation.
